# Improved Protective Effect of Umbilical Cord Stem Cell Transplantation on Cisplatin-Induced Kidney Injury in Mice Pretreated with Antithymocyte Globulin

**DOI:** 10.1155/2016/3585362

**Published:** 2016-01-05

**Authors:** Željka Večerić-Haler, Andreja Erman, Anton Cerar, Helena Motaln, Katja Kološa, Tamara Lah Turnšek, Snežna Sodin Šemrl, Katja Lakota, Katjuša Mrak-Poljšak, Špela Škrajnar, Simona Kranjc, Miha Arnol, Martina Perše

**Affiliations:** ^1^Department of Nephrology, University Medical Centre Ljubljana, 1000 Ljubljana, Slovenia; ^2^Institute of Cell Biology, Faculty of Medicine, University of Ljubljana, 1000 Ljubljana, Slovenia; ^3^Institute of Pathology, Medical Experimental Centre, Faculty of Medicine, University of Ljubljana, Zaloška 4, 1000 Ljubljana, Slovenia; ^4^National Institute of Biology, 1000 Ljubljana, Slovenia; ^5^Jožef Štefan International Postgraduate School, 1000 Ljubljana, Slovenia; ^6^Department of Rheumatology, University Medical Centre Ljubljana, 1000 Ljubljana, Slovenia; ^7^Lek Pharmaceuticals d.d. Ljubljana, 1000 Ljubljana, Slovenia; ^8^Department of Experimental Oncology, Institute of Oncology, 1000 Ljubljana, Slovenia

## Abstract

Mesenchymal stem cells (MSCs) are recognised as a promising tool to improve renal recovery in experimental models of cisplatin-induced acute kidney injury. However, these preclinical studies were performed on severely immunodeficient animals. Here, we investigated whether human umbilical cord derived MSC treatment could equally ameliorate acute kidney injury induced by cisplatin and prolong survival in mice with a normal immune system and those with a suppressed immune system by polyclonal antithymocyte globulin (ATG). We demonstrated that ATG pretreatment, when followed by MSC transplantation, significantly improved injured renal function parameters, as evidenced by decreased blood urea nitrogen and serum creatinine concentration, as well as improved renal morphology. This tissue restoration was also supported by increased survival of mice. The beneficial effects of ATG were associated with reduced level of inflammatory protein serum amyloid A3 and induced antioxidative expression of superoxide dismutase-1 (SOD-1), glutathione peroxidase (GPx), and hem oxygenase-1 (HO-1). Infused MSCs became localised predominantly in peritubular areas and acted to reduce renal cell death. In conclusion, these results show that ATG diminished in situ inflammation and oxidative stress associated with cisplatin-induced acute kidney injury, the effects that may provide more favourable microenvironment for MSC action, with consequential synergistic improvements in renal injury and animal survival as compared to MSC treatment alone.

## 1. Introduction

Over the last decade, mesenchymal stem cells (MSCs) were shown to be one of the promising tools to treat acute kidney injury (AKI) in various animal models, including cisplatin-induced nephrotoxicity [1–3]. It has been shown that MSC transplantation markedly improved animal survival as well as functional and morphological parameters of cisplatin-induced AKI [4–8]. In accordance with the discovery that MSCs can produce a number of immunomodulatory molecules and secrete various soluble factors that reduce apoptosis and promote mitosis, it is believed that these properties are responsible for beneficial outcome of MSC therapy in AKI [9]. Recently it has been demonstrated that MSC secreted factors have beneficial effects in various immune disorders, including transplantation rejection and graft-versus-host diseases (GVHD) [9–11]. MSCs from human umbilical cord have been successfully used in cell therapies as immunoregulators for the treatment of GVHD in humans [12] and in various immunocompetent animal models without the use of immunosuppressants for the treatment of targeted diseases [13], including cisplatin nephrotoxicity [2]. Nevertheless, most of the studies that evaluated beneficial effects of human MSCs in cisplatin-induced AKI have been so far performed on immunocompromised animals [4, 6].

It is known that both innate and adaptive immune systems are important contributor to the pathogenesis of cisplatin-induced AKI and can significantly affect the extent of cisplatin nephrotoxicity [14]. For instance, severely immunocompromised animals are susceptible to cisplatin nephrotoxicity [4], while mice without T lymphocytes, CD4^+^, or CD8^+^ T cells are protected against cisplatin nephrotoxicity [15]. However, it is not known whether compromised immune system of animals could interfere with the effects of MSC therapy.

Antithymocyte globulin (ATG) has been successfully used for decades in clinical transplantation due to its immunosuppressive role in GVHD and solid organ rejection. The immunosuppressive effects of ATG in clinical transplantation have been mostly attributed to its ability to reduce circulating T lymphocytes. However, recent data suggest that ATG has many pleiotropic immunomodulatory properties including inhibition of B lymphocytes, dendritic and natural killer cells as well as modulation of surface adhesion molecules and chemokine receptor expression [16]. Thus, immunosuppression by ATG can be good alternative to evaluate the effect of compromised immune system on MSC therapy in cisplatin-induced AKI. T cell depletion in a murine model of ischemic injury using different antibodies to T cell epitopes has been shown to be effective in ameliorating the course of experimental intestinal ischemic injury in mice [17]. Whether ATG alone or in combination with MSCs can affect susceptibility to cisplatin injury has not so far been established.

To evaluate the interference of compromised immune system on MSC therapy in cisplatin-induced AKI, we analyzed morphological, functional, oxidative, and inflammatory alterations in the kidney of mice with a normal immune system and those with a suppressed immune system by ATG. Our aim was to evaluate both the separate and combined effects of ATG and MSCs on cisplatin-induced acute nephrotoxicity.

## 2. Materials and Methods

### 2.1. Isolation and Characterization of MSCs

Umbilical cord (UC) derived MSCs were isolated from Wharton's jelly according to standard protocol [18] in the study approved by the National Ethics Committee (Document number 134/01/11). Umbilical cords were collected at cesarean section (37–41 weeks) upon obtained informed consent, and isolated MSC clones were cultured in Dulbecco's medium (DMEM; 5921; Sigma-Aldrich) with 10% fetal bovine serum (FBS; PAA Lab), supplemented with 100 U penicillin (PAA Lab), 1,000 U streptomycin (PAA Lab), 2 mM L-glutamine (PAA Lab), Na-pyruvate (Gibco), and nonessential amino acids (Sigma-Aldrich). MSC clones were characterized for CD13^+^, CD29^+^, CD44^+^, CD73^+^, CD90^+^, CD105^+^, CD14^−^, CD34^−^, CD45^−^, and HLA-DR^−^ surface marker expression and osteogenic, chondrogenic, and adipogenic differentiation as recommended [19]. Three MSC clones (UC-MSC 10, female; UC-MSC 8, male; and UC-MSC 5, female) with the highest proliferation potential and homogenous spindle-like morphology were used in further animal experiments. The mix of all three MSC clones was used for every animal injection. The reason for mixing three different MSC lines prior to every injection remains in their proven clonal heterogeneity.

### 2.2. Fluorescence Microscopy and Detection of Labeled MSCs

Detection and intrarenal localization of MSCs labeled with red fluorescence cell dye DiI (Invitrogen, Carlsbad, CA, USA) was analyzed by fluorescence microscope Nikon Eclipse TE 300 (Amstelveen, Netherlands). Firstly, MSCs in culture were labeled with lipophilic dye DiI (5 *μ*L/mL in medium) for 30 minutes at 37°C. After labeling, cells were rinsed with fresh medium four times. Labeling efficacy was assessed to be >97%. Viability evaluated by Trypan blue exclusion was >96%. Labeled MSCs were then infused in mice 24 hours after cisplatin administration and at day 4 mice were sacrificed and tissue samples were fixed in 3% paraformaldehyde in phosphate-buffered saline (PBS) for 2 hours. After overnight rinsing in 30% sucrose, tissue samples were frozen and cut into 5 *μ*m thick cryosections, which were thawed, washed in PBS, and mounted in antibleaching mounting medium Vectashield with 4′,6-diamidino-2-phenylindole (DAPI) (Vector Laboratories, Burlingame, CA, USA) to stain DNA. Quantitation of DiI labeled MSCs was performed on tissue sections of each animal in 3 nonoverlapping fields at 100x magnification.

### 2.3. Animals and Experimental Protocol

All procedures involving animals were approved by the National Ethical Committee and the Administration of the Republic of Slovenia for Food Safety, Veterinary and Plant Protection (Permit number 34401-54/2012/5). Animal care and treatment were conducted in accordance with the institutional guidelines and international laws and policies (Directive 2010/63/EU on the protection of animals used for scientific purposes).

Experiment was carried out on 8–12-week-old male BALB/cOlaHsd mice (Harlan, Italy; *n* = 80). Mice were housed in medical experimental center animal facility under controlled conditions of temperature (22 ± 1°C), humidity (55 ± 10%), and 12 h/12 h light-dark cycle (7 a.m.–7 p.m. light) with unlimited access to laboratory diet (Harlan Teklad 2018) and water. During the experiment, the animals were housed individually in open bar cages (Ehret, Germany; 375 cm^2^ floor space) on bedding (Lignocel 3/4 Germany) enriched with nesting material (paper towels).

Models of AKI were induced by intraperitoneal (ip) injection of a single dose of 17 mg/kg of cisplatin (Pliva-Teva, Croatia) dissolved in 0.9% saline solution (1 mg/2 mL). The dosage of cisplatin was based on literature analysis and results of our preliminary experiment, showing renal function impairment, as indicated by significantly increased levels of blood urea nitrogen (BUN) and creatinine as well as marked histologic changes in animals at day 4 after cisplatin application.

**Figure 1:**
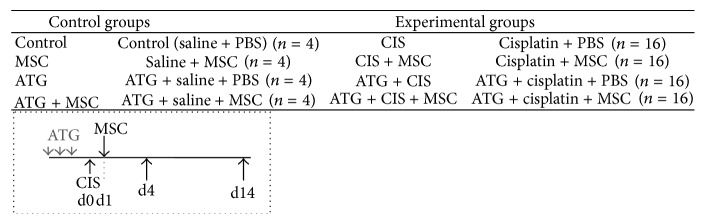
Shematic representation of the protocol and all control and experimental groups.

Antimouse antithymocyte globulin (ATG, Fresenius-Biotech-GMBH, Germany) was used as pretreatment to induce partial immunotolerance in mice. ATG is a polyclonal purified IgG fraction of sera from rabbits immunized with mouse thymocytes. ATG was provided as solution of 15.2 mg/mL and was injected at a dosage of 1.8 mg ip 3 consecutive days before cisplatin application (Figure 1). MSCs were injected intravenously 24 hours after cisplatin administration (day 1). Mice received 5 × 10^5^ MSCs at sixth passage in overall 0.2 mL of PBS medium. At day 4 after cisplatin application, mice were sacrificed with CO_2_. Blood was taken by heart puncture and kidneys were excised, weighed, and collected for histology, immunohistochemistry, and mRNA expression analyses. At autopsy also all visible changes and other solid organs (lung, liver, intestine, heart, spleen, and testicles) were also weighed, collected, and analyzed in order to evaluate stem cell trafficking and exclude potential host versus graft disease.

Half of the animals for each experimental group (*n* = 8) were euthanized at day 4 and the other half of the animals for each experimental (*n* = 8) and each control group (*n* = 4) were used to assess survival for at least 14 days. During the experiment, mice were carefully observed a few times daily and when clinical signs of mice reached humane end points (point of nonreturn; body temperature drop, ataxia), mice were euthanized to minimize their suffering.

Mice in all groups were treated in the same manner and have undergone the same procedures. For instance, mice received saline instead of cisplatin or 0.2 mL of PBS instead of MSC. The animals were divided into 4 control groups (*n* = 4 per group) and 4 experimental groups (*n* = 16 per group) as shown in Figure 1.

### 2.4. Serum Measurements

On the day of euthanasia, blood was immediately collected by cardiac puncture and serum levels of blood urea nitrogen (BUN) and creatinine were measured using a blood chemistry analyzer.

### 2.5. Histological Evaluation

For renal histology, 4 *μ*m-thick paraffin kidney sections were stained with periodic acid-Schiff (PAS). For intestine histology, 4 *μ*m-thick paraffin sections were stained with Kreyberg-Jareg stain. Histology was evaluated by two pathologists in blinded fashion. The score of AKI was assessed in a double-blind fashion in 10 nonoverlapping fields (for each animal) using 400x magnification.

The severity of renal toxicity, including damage to the glomeruli, tubules, interstitium, and renal blood vessels, was assessed as described previously [20].

The criteria of a semiquantitative scoring scale of 0–5 were as follows:

- 0 is normal tubules, glomerulus, interstitium, and vessels.
- 1 is scant number of tubular epithelial cells showing minimal degeneration, mild tubular dilatation, small number of proteinaceous casts, no regeneration, and no definitely significant necrosis or apoptosis and no changes in the glomerulus, interstitium, and vessels.
- 2 is <25% of tubular epithelial cells showing mild degeneration (large cytoplasmic vacuoles, a few hyaline droplets in the cytoplasm), mild degree of tubular dilation and proteinaceous casts, slight change in tubular brush border loss, acute tubular necrosis in individual cell or small group of cells, a few apoptotic cells, and no regeneration and no changes in the glomerulus, interstitium, and vessels.
- 3 is 25%–50% of tubular epithelial cells showing moderate degeneration (multiple large-sized vacuoles, multiple foci of hyaline droplets), mild regeneration, moderate tubular brush border loss, moderate acute tubular necrosis in small group of tubules, and increased number of apoptotic cells; little involvement of mild glomerular vacuolization; and no changes in the interstitium and vessels.
- 4 is 51%–75% of tubular epithelial cells showing extensive moderate degeneration; moderate regeneration; severe tubular brush border loss; severe acute tubular necrosis; and a large number of apoptotic cells, with apoptotic bodies in clusters of tubules, and little involvement of mild glomerular vacuolization and interstitial lymphocytic infiltration.
- 5 is >75% of tubular epithelial cells showing severe degeneration, regeneration, severe tubular brush border loss, acute tubular necrosis, and a large number of apoptotic cells with numerous apoptotic bodies; mild involvement of glomerular injury (vacuolization, mesangial cell proliferation, and increase in mesangial matrix) and interstitial lymphocytic infiltration; and no significant changes in the vessels.

### 2.6. Immunostaining of Caspase 3

Tissue pieces were fixed in 4% formaldehyde overnight. After embedding in paraffin and deparaffinization through graded alcohols, 4 *μ*m thick tissue sections were microwave-heated for 5 min using 10 mM citrate buffer (pH 6.0) for antigen retrieval. Endogenous peroxidase activity was blocked by incubating the sections in 3% H_2_O_2_ in methanol for 15 min. After permeabilization in 0.4% Triton X-100 in PBS for 5 min, sections were washed in PBS and blocked with 5% BSA in PBS for 1 hour at 37°C. Samples were then incubated with the primary antibody against caspase 3 (Abcam; rabbit polyclonal, 1/300) overnight at 4°C. As negative controls, the primary antibody was replaced with PBS buffer. After washing in PBS, biotinylated swine anti-rabbit antibody (Dako; 1/200) was applied for 1 hour at 37°C, followed by incubation with ABC complex/HRP (Dako) for 30 min at room temperature. After the standard DAB (Sigma) development procedure, sections were stained in Mayer's haematoxylin, dehydrated, mounted in Depex (Agar), and examined with light microscope. Each tissue sample stained for caspase 3 was viewed and scored by two observers in a double-blind fashion. Quantitation of caspase 3 positive cells was performed in 10 nonoverlapping fields at 200x magnification and the average value of two observations was calculated and statistically analyzed.

### 2.7. Electron Microscopy

Examination of ultrastructural changes of renal proximal tubule cells was performed by transmission electron microscopy. Pieces of kidney tissue were fixed in a mixture of 4% paraformaldehyde and 2% glutaraldehyde in 0.2 M cacodylate buffer (pH 7.3) for 3 hours at 4°C. After rinsing in 0.33 M sucrose in the same buffer and postfixation in 1% OsO_4_ for 1 hour, tissue samples were dehydrated through ascending grades of ethanol and embedded in Epon (Serva Electrophoresis, Heidelberg, Germany). Ultrathin sections were stained with uranyl acetate and lead citrate and examined with a Jeol 100 CX (Tokyo, Japan) electron microscope.

### 2.8. RNA Isolation and Quantitative PCR Analysis

Equivalent parts of the mouse kidney were homogenized in lysis buffer with TissueLyser LT (Qiagen, Hilden, Germany) and total RNA was isolated from homogenate using RNeasy Micro Kit (Qiagen, Hilden, Germany) following the manufacturers' instructions. The purity and amount of RNA were determined by measuring the OD at a ratio of 260 to 280 nm. cDNA was generated from 1 *μ*g of total RNA using the Reverse Transcription System (Promega, WI, USA) with oligo(dT) primers, and qPCR was performed with StepOne Real-time PCR System (A&B Applied Biosystems, Life Technologies, Darmstadt, Germany) in triplicate using 200 nM of specific primers (see Table 1), 10 ng cDNA, and SYBR Select Master Mix (Life Technologies, Carlsbad, CA). Melting curve for each reaction was performed and confirmed only one product. Data analysis was done using 2ddCt method with normalization to the level of L32. Primer sets are shown in Table 1.

### 2.9. Statistical Analysis

The results are expressed as mean ± SEM. Statistical comparison of the data was performed using the *t*-test for comparison between two groups or multivariate analysis of variance and the post hoc Tukey range tests for comparison of more than two groups. Survival data were analyzed using the Kaplan-Meier test. Statistical significance level was defined as *p* < 0.05. Statistical analyses were performed using the Statgraphics Centurion XVI (version 16.1.11) and SPSS software (IBM SPSS statistics, version 22.0).

## 3. Results

### 3.1. Effect of ATG Pretreatment

Results show that ATG caused selective immunosuppression with depletion of lymphocytes, eosinophils, and basophils without significant influence on other parameters of differential white cell spectrum or on platelet or erythrocyte count (Table 2). Interestingly, ATG pretreatment had no other effects on clinical appearance of mice or their body weight, kidney morphology, or function (Table 3).

### 3.2. MSCs Distribution from Results of Fluorescence Microscopy Analysis

**Figure 2:**
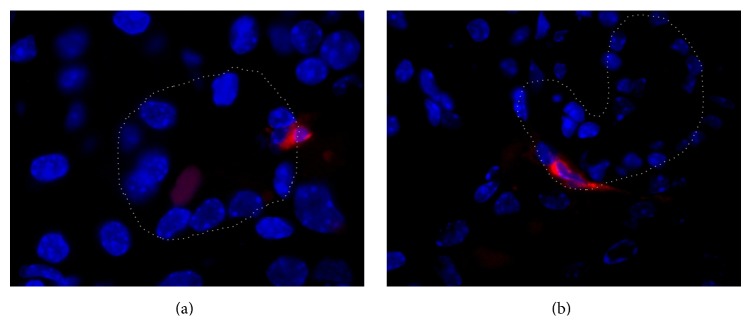
Representative images of kidney tissue in mice with cisplatin-induced AKI treated with DiI labeled MSCs (day 4). (a) DiI labeled MSCs (red fluorescence) in peritubular area. Original magnification: ×1000. (b) DiI labeled MSCs (red fluorescence) within proximal tubule epithelium. Original magnification: ×1000. Nuclei are stained with DAPI (blue fluorescence). Basal lamina of proximal tubule is marked with a gray line.

Labeled MSCs were detected in different organs (lungs, liver, intestine, and kidney). In kidney, MSCs were found predominantly in peritubular regions; occasionally, they were localized within the proximal tubule epithelium and never in glomeruli (Figure 2). Distribution of MSCs in analyzed tissues was as follows: in lungs 10 MSCs, in liver 136 MSCs, in intestine 3 MSCs, and in kidney 4 MSCs per field (magnification ×100).

### 3.3. MSC Transplantation Reduced Cisplatin-Induced Mortality in Mice Pretreated with ATG

**Figure 3:**
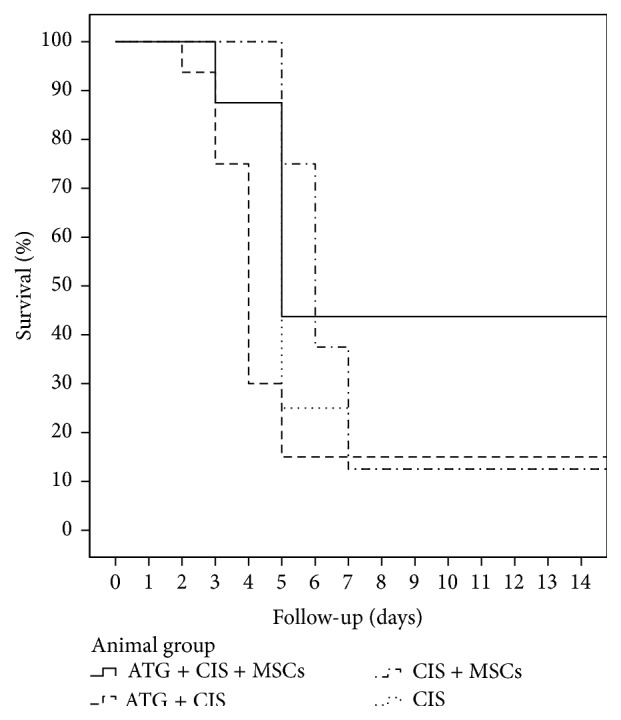
Schematic representation of survival curves in BALB/cOlaHsd cisplatin treated mice. The survival was estimated by Kaplan-Meier statistical analysis. Mice pretreated with ATG had an increased survival after MSCs treatment during their follow-up (log rank *p* = 0.012).

Administration of cisplatin resulted in reduction of body weights in all cisplatin treated groups. Mice with cisplatin-induced AKI had the lowest survival, while mice treated with single injection of MSCs after ATG immunosuppression had the highest survival among cisplatin treated groups (43.5% survival at day 30). Survival data and curves of cisplatin treated mice given MSCs or/and ATG are shown in Figure 3.

### 3.4. MSC Treatment Improved Renal Function and Morphology in Cisplatin-Induced Mice Pretreated with ATG

**Figure 4:**
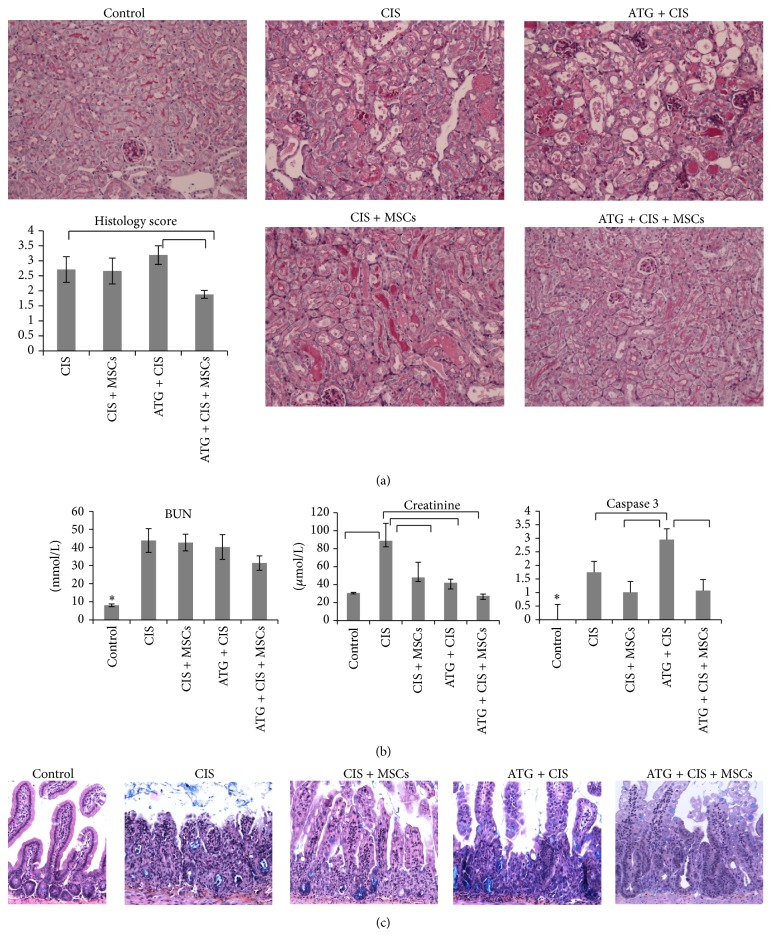
MSC treatment after ATG immunosuppression improves renal function and morphology as well as intestine morphology in BALB/cOlaHsd mice 4 days after cisplatin administration. (a) Representative micrographs of renal histology of healthy control mice and of mice treated with cisplatin and saline or MSCs and/or ATG (original magnification: ×200, periodic acid-Schiff (PAS) staining). (b) Serum concentration of blood urea nitrogen (BUN) and creatinine and caspase 3 score. Graphical results are expressed as mean ± SEM; ANOVA followed by Duncun multicomparison test: ^*∗*^ *p* < 0.05 control versus all other groups. (c) Representative micrographs of intestine of healthy control mice and of mice treated with cisplatin and saline or MSCs and/or ATG (original magnification: ×200, Kreyberg-Jareg staining).

In contrast to all control groups, mice treated with cisplatin showed morphological and functional changes in kidneys 4 days after treatment as demonstrated by a significant increase in histology score, serum levels of BUN (*p* < 0.0001), and creatinine (*p* < 0.0001) (Table 3, Figures 4(a) and 4(b)).

Improvement of both renal morphology and function was found only in mice treated with both ATG and MSCs. Cisplatin mice treated with ATG and MSCs had significantly lower relative kidney weight (*p* = 0.006), histology score (*p* = 0.005), serum creatinine levels (27.5 ± 1.91 versus 42.0 ± 3.96; *p* = 0.015), and nonsignificantly lower BUN (31.34 ± 3.99 versus 40.26 ± 6.84; *p* = 0.2) in respect to mice treated with ATG and cisplatin alone (Table 3, Figures 4(a) and 4(b)).

At day 4 after cisplatin administration, kidneys of mice treated with ATG showed tubular lesions consisting of multiple large-sized vacuoles, multiple foci of luminal cell debris and hyaline casts, moderate tubular brush border loss, and moderate acute tubular necrosis in small group of tubules. Changes were seen in proximal as well as distal tubules, while glomerular changes were not detected. Mice treated with MSCs showed less damage in the kidney, but the difference was not significant. On the other hand, treatment with MSCs after ATG immunosuppression resulted in significant improvement. In particular, tubular epithelial cells showed minimal degeneration with rare necrosis and apoptosis, and hyaline casts were absent (Figure 4(a)).

Interestingly, although treatment with ATG or MSCs alone had no significant improvement on morphology of cisplatin-damaged kidney, serum creatinine concentrations (but not BUN) were reduced in both instances. It is known that serum concentration of creatinine is not always in strict correlation with glomerular filtration. Intestinal disturbances caused by some chemotherapeutics, including cisplatin, could raise serum creatinine concentration. Thus, processes other than a decline in renal function need to be excluded if BUN concentration remains unchanged.

Based on observation of this discrepancy and the presence of bloody diarrhea in cisplatin treated mice, the intestine was morphologically evaluated. Histological analyses revealed that cisplatin administration resulted in acute inflammation of the intestine with moderate to severe injury of crypts evidenced by crypt abscesses, cryptitis, and crypt dilatation. The villi shortened or in some cases completely disappeared (Figure 4(b)). It was found that treatment with MSCs or ATG reduced intestinal injury, while treatment with MSCs after ATG immunosuppression resulted in markedly reduced injury and intensive regeneratory activity in the intestine (Figure 4(b)). Thus, MCS treatment improved intestine morphology as well.

### 3.5. Kidney Ultrastructure

**Figure 5:**
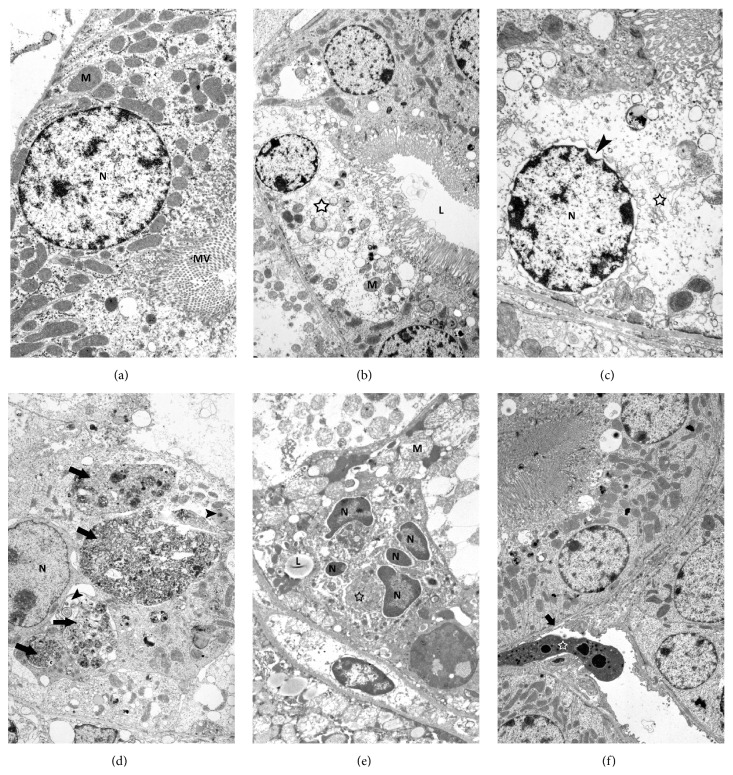
Kidney ultrastructure. (a) Ultrastructure of epithelial cell with large and rounded nucleus (N), numerous mitochondria (M), and pronounced microvilli (MV). (b) Necrotic epithelial cell (asterisk) with typical ultrastructural signs of necrosis such as electron pale and disintegrated cytoplasm, disrupted microvilli, and few mitochondria (M). L-lumen of proximal tubule. (c) Details of necrotic epithelial cell with strongly dilated perinuclear space (arrowhead), ruptured Golgi apparatus (asterisk), and highly demolished cytoplasmic integrity with hardly recognizable structures. (d) Epithelial cell with no brush border on apical surface, multilamellar bodies (arrowheads), and extremely large autophagic vacuoles (arrows) and fulfilling the entire cytoplasm. N-nucleus. (e) Apoptotic epithelial cell with condensed chromatin in nuclear fragments (N), lipid droplets (L), disappeared microvilli, and electron dense and condensed cytoplasm (asterisk). (f) Epithelial cells of proximal tubule with normal ultrastructure. Under basal lamina (arrow) of tubule, peritubular cell (asterisk) of connective tissue with typical signs of apoptosis such as cytoplasm condensation and chromatin fragmentation and condensation is present. Representative TEM images of kidney samples taken from control mice (a), mice receiving ATG and cisplatin (b–d), and mice receiving MSCs after ATG and cisplatin (e, f). Original magnifications: ×4400 (a), ×2600 (b), ×5800 (c), ×2600 (d), ×4400 (e), and ×2000 (f).

Results of transmission electron microscopy analysis confirmed histological results and revealed that cisplatin administration resulted in markedly changed ultrastructure of tubular epithelial cells, which showed typical characteristics of necrotic cell death such as ruptured apical plasma membrane, disrupted microvilli, swollen nucleus with disintegrating chromatin, dilatation of the endoplasmic reticulum, Golgi apparatus and perinuclear space, and vacuolized and disintegrated cytoplasm eventually (Figures 5(b) and 5(c)). The amount of mitochondria was strongly reduced in necrotic cells, but their structure was intact until the very late stages of necrosis. Beside numerous necrotic epithelial cells of proximal tubules also cells with many autophagic vacuoles and multilamellar bodies were found indicating intense autophagic activity in these cells (Figure 5(d)). Mice treated with MSCs after ATG immunosuppression showed less injured ultrastructure of proximal tubular epithelial cells with only few necrotic cells and cells with intense autophagic activity. Beside them, individual apoptotic epithelial cells and peritubular cells of connective tissue were detected (Figures 5(e) and 5(f)).

### 3.6. Effect of ATG and MSCs on Caspase 3 Staining and Gene Expression of Inflammatory and Oxidative Proteins

In all experimental groups, the number of caspase 3 positive tubular cells was low (on average 0 ± 0.57 positive cells per field, magnification ×200), while treatment with cisplatin resulted in a significantly increased number of caspase 3 positive cells (*p* = 0.01, Figure 4(b)). The highest caspase 3 score was found among cisplatin treated groups where mice were pretreated with ATG (*p* < 0.05). Interestingly, MSCs significantly decreased caspase 3 score in cisplatin mice pretreated with ATG (*p* = 0.04).

SAA3 is an important inflammatory marker and acute phase protein in mice. As expected, cisplatin treatment resulted in a significant increase in SAA3 expression (5-fold increase compared to control). Interestingly, MSC treatment had no beneficial effects on SAA3 expression (*p* = 0.3), while ATG pretreatment significantly reduced expression of SAA3 (*p* = 0.002). Cisplatin increased also renal expression of cytokine ICAM-1, while it had no effects on the expression of TGF-*β* or IL-1*β*.

Cisplatin treatment significantly reduced expression of the main oxidative enzymes in kidney, such as CAT, SOD1, and SOD2, but had no significant influence on GPx. The upregulation of ICAM-1 or downregulation of CAT, SOD1, and SOD2 was not restored by ATG or MSC treatment.

**Figure 6:**
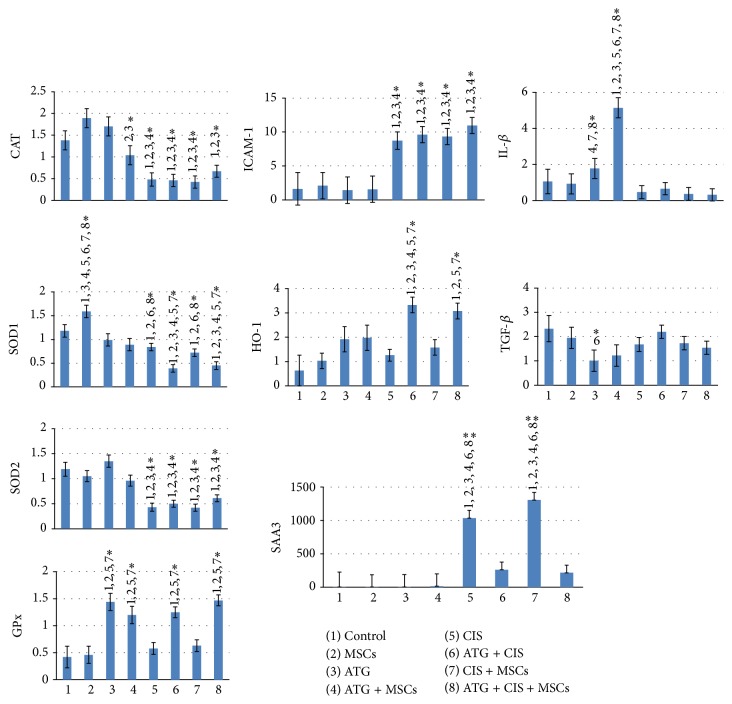
Gene expression of inflammatory and oxidative proteins in kidneys of 8–12-week-old male BALB/cOlaHsd mice after cisplatin-induced AKI treated with ATG and/or MSCs. Bars represent alterations in expression of cytokines, inflammatory, and oxidative proteins as measured by RT-PCR in kidney tissues of sacrificed mice. Results are expressed as mean ± SEM. ANOVA followed by Duncun multicomparison test: ^*∗*^ *p* < 0.05; ^*∗∗*^ *p* < 0.001; SAA3: serum amyloid A3, HO-1: heme oxygenase-1, GPx: glutathione peroxidase, CAT: catalase, SOD-1: superoxide dismutase-1, SOD-2: superoxide dismutase 2, IL-1*β*: interleukin 1*β*, TGF-*β*: transforming growth factor *β*, and ICAM-1: intercellular adhesion molecule 1.

ATG pretreatment attenuated the mRNA expression of SAA3 and accelerated expression of HO-1 in kidneys of cisplatin treated mice. ATG pretreatment resulted also in significant increase in GPx expression in kidneys of both healthy and cisplatin treated mice. In contrast, MSC treatment had no significant beneficial effect on altered expression of measured proteins induced by cisplatin. Likewise, MSC or ATG treatment alone had no influence on the expression pattern of other proteins measured in kidneys of healthy mice. Gene expression of inflammatory and oxidative proteins in kidneys is shown in Figure 6.

## 4. Discussion

The present study demonstrates for the first time that suppression of an immune system by ATG significantly improves beneficial effects of MSC transplantation in cisplatin-induced AKI in mice. Results demonstrate that treatment with MSCs alone resulted in tendency but not significant improvement of functional and morphological changes in cisplatin-induced AKI, while administration of MSCs after ATG immunosuppression resulted in amelioration of renal function and in increased survival of mice. Mice treated with MSCs after ATG immunosuppression had restored structure of kidney tissue on histologic and ultrastructural level. Only mild lesions, such as mild degeneration and slight change in tubular brush border with rare apoptotic or necrotic cells, were observed in renal tubular epithelial cells.

It is believed that one of MSCs' beneficial effects is related to impaired apoptosis [4] that was in our case observed in cisplatin-induced AKI, but only when MSC treatment followed ATG immunosuppression. Our results showed that cisplatin treatment resulted in various forms of cell deaths [21, 22], such as apoptosis, necrosis, and even autophagic cell death in epithelial cells of proximal tubules. In cisplatin treated mice, intense necrotic and autophagic cellular appearances were observed, while in mice treated with ATG and MSCs necrotic and particularly autophagic cell features were less frequent. Thus, combination of ATG and MSC treatment may have preventive effects against different forms of cell death, not only apoptosis.

Various mechanisms are involved in cisplatin nephrotoxicity, including oxidative stress and inflammation [14]. It was demonstrated that T cell-deficient (nu/nu) mice and CD4^−/−^ and CD8^−/−^ cell-deficient mice had marked attenuation in renal dysfunction, morphology, and survival [15]. Likewise, administration of T regulatory lymphocytes (Treg cells, CD4^+^CD25^+^) has ameliorated cisplatin nephrotoxicity in mice [23]. Immunosuppressive effects of both ATG and MSCs (i.e., inhibition of the activation of CD4^+^ and CD8^+^ T cells and induction of Treg cells) [9, 16] may thus act protectively against cisplatin nephrotoxicity and reduce inflammation and oxidative stress in injured tissue.

To evaluate immunosuppressive efficacy of ATG and MSCs on cisplatin-induced AKI, one of the most known proteins produced in the acute phase of inflammation, serum amyloid A3 protein (SAA3), was assessed. Many acute phase proteins are upregulated early after renal failure as a consequence of inflammatory response in AKI [24]. SAA3 has several roles, including the recruitment of immune cells to inflammatory sites [25]. As mentioned above, a variety of activated immune cells accumulating in the kidney have different impact on the course of cisplatin-induced AKI and can either repair or further aggravate the injury. Accumulation of T lymphocytes, particularly CD4^+^ and CD8^+^ cells, [15] has been shown to aggravate the injury, whereas accumulation of Treg cells has been associated with amelioration of the injury [23]. In contrast, depletion of neutrophils had no protective effect against cisplatin nephrotoxicity [26].

Nevertheless, our results show that ATG significantly suppressed SAA3 in cisplatin-induced AKI. It was shown that ATG has ability to reduce infiltration of CD3^+^, CD4^+^, and CD8^+^ lymphocytes as well as neutrophils in the injured tissue [17]. Significantly the reduced number of lymphocytes in the blood and suppressed SAA3 in cisplatin treated kidney in our study suggests that ATG inhibited infiltration of inflammatory cells in the kidney, which resulted in amelioration of AKI and reduced cell death.

Cisplatin-induced AKI is associated with the release of many soluble mediators and activation of adhesion molecules from activated or injured kidney cells (such as ICAM-1, TGF-*β*, TNF-*α*, IL-1*β*, and RANTES), which further attract and activate leukocytes to sites of injury [27]. In animal models of cisplatin nephrotoxicity, blockade or deletion of some of the inflammatory cytokines, such TNF-*α* or ICAM-1, reduced the severity of renal injury [27–29].

It was reported that ATG and MSCs have immunomodulatory effects, ATG by modulating surface adhesion molecules such as ICAM-1 [16] and MSCs by producing molecules such as TGF-*β* and HO-1 [9]. Surprisingly, in our study MSCs administration had no significant effect on the expression of TGF-*β* or HO-1 and ATG pretreatment with or without MSCs administration did not alter TGF-*β*, IL-1*β*, or ICAM-1 expression in the kidney of cisplatin treated mice, suggesting that there are other mechanisms implicated in its protective actions.

To evaluate oxidative mechanisms of ATG alone and in combination with MSCs, we assessed expression of antioxidant enzymes in renal tissue. In agreement with others [27, 30], cisplatin decreased expression of antioxidative enzymes SOD and CAT and increased expression of the adhesion molecule ICAM-1. Again, MSCs administration alone had no significant effect on the expression of antioxidant enzymes, while ATG pretreatment significantly affected expression of SOD1, GPx, and HO-1. Several studies have shown that induction of GPx, which has dual antioxidative function in the cell, and HO-1, which confers anti-inflammatory response, has protective effect in cisplatin nephrotoxicity [31, 32]. Agents with the ability of antioxidant enzymes activity like GPx ameliorated cisplatin-induced kidney damage and improved renal function [31], while the deletion of HO-1^−/−^ gene or even inhibition of HO-1 significantly worsened both structural and functional parameters of renal injury [33]. Taken together, in our study, induction of GPx and HO-1 by ATG may be implicated in amelioration of cisplatin nephrotoxicity.

On the other hand, MSCs administration alone had no influence on white cells in the blood or the expression of SAA3 or other oxidative and inflammatory proteins (ICAM-1, HO-1, IL-1*β*, and TGF-*β*) in the kidney. SAA3 proved to be an excellent marker of inflammation, highly inducible by cisplatin, with ATG greatly and significantly attenuating its gene expression levels, both in presence and in absence of MSCs. This is in line with our previous mouse study, where SAA3 served as a responsive inflammatory marker for liver, bladder wall, and urothelial mRNA expression, as well as serum levels induced following intraperitoneal inoculation of uropathogenic* Escherichia coli* [34].

Since we used lethal murine model of AKI with multiorgan failure, it is very likely that single administration of MSCs was insufficient to cope with extremely harmful environment. As demonstrated, cisplatin caused not only acute nephrotoxicity but also acute severe intestinal inflammation with bloody diarrhea, which affected creatinine levels and very likely also other molecules. Multiorgan failure results in altered hemodynamic state and renal vascular autoregulation, which further worsens renal function. Such models introduce much complexity in treatment strategy, especially in regard to systemic treatment and cell based therapies. It was suggested that one of the major problems influencing the efficacy of stem cell therapy is the poor MSCs survival following transplantation. This could at least partly be attributed to insufficient resistance of transplanted stem cells to oxidative and inflammatory stresses at the injured sites. Although MSCs may require activation by signals from a proinflammatory environment to modulate the activity of surrounding cells [35], the overload of proinflammatory cytokines may be harmful for MSCs and impair their functionality [36]. In addition, in vitro study has shown that MSCs become immunosuppressive not earlier than after they are activated by blood CD14^+^ monocytes via IL-1*β* [35]. Results of our study show that neither ATG nor MSCs alone affected IL-*β* expression in the kidney, while treatment with both ATG and MSCs resulted in significantly increased expression of this cytokine in healthy kidney. These results indicate that ATG pretreatment not only may reduce inflammation but also resulted in stimulation or activation of MSCs immunosuppressive effects.

Taken together, our study shows that ATG pretreatment in MSC therapy, directed against cisplatin-induced AKI, significantly improved renal status as observed by morphological and functional parameters of injured kidney as well as prolonged lifespan of mice. The observed positive effects were due to reduced inflammation and cell death, caused by cisplatin, as measured also by immunological mediators and oxidative enzymes. ATG pretreatment created favourable immunological environment in immunocompetent mice, by both diminishing the level of inflammation and oxidative stress at the site of injury that may allowed further immunomodulatory activities of MSCs. As reviewed by Motaln and Lah [37, 38] and others, MSCs secrete various chemokines in response to their environment and can on the other hand also respond to them by differentiating into various cells, depending on microenvironmental cues. This notion adds to beneficial effects on MSC therapy. The results of this study further suggest that altering immunologic microenvironment in uncompromised environment in patients by ATG or other agents may improve human MSC transplantation efficacy and therapeutic value.

## Figures and Tables

**Table 1:** Mouse primers used for QPCR analysis of inflammatory cytokines and oxidative stress enzyme expression in renal tissue.

Parameter	Primer
IL-1*β*	F: 5′-CAACCAACAAGTGATATTCTCCATG-3′
	R: 5′-GATCCACACTCTCCAGCTGCA-3′

ICAM-1	F: 5′-CAATTTCTCATGCCGCACAG-3′
	R: 5′-AGCTGGAAGATCGAAAGTCCG-3′

HO-1	F: 5′-GGTGATGGCTTCCTTGTACC-3′
	R: 5′-AGTGAGGCCCATACCAGAAG-3′

SOD1	F: 5′-CCAGTGCAGGACCTCATTTT-3′
	R: 5′-CACCTTTGCCCAAGTCATCT-3′

SOD2	F: 5′-GGCCAAGGGAGATGTTACAA-3′
	R: 5′-GAACCTTGGACTCCCACA-3′

CAT	F: 5′-CCGACCAGGGCATCAAAA-3′
	R: 5′-GAGGCCATAATCCGGATCTTC-3′

GPx	F: 5′-CCACCGTGTATGCCTTCTCC-3′
	R: 5′-GATCGTGGTGCCTCAGAGAG-3′

TGF*β*	F: 5′-GACCGCAACAACGCCATCTA-3′
	R: 5′-GGCGTATCAGTGGGGGTCAG-3′

SAA3	F: 5′-TGC CAT CAT TCT TTG CAT CTT GA-3′
	R: 5′-CCG TGA ACT TCT GAA CAG CCT-3′

SAA3: serum amyloid A3, HO-1: heme oxygenase-1, GPx: glutathione peroxidase, CAT: catalase, SOD-1: superoxide dismutase-1, SOD-2: superoxide dismutase 2, IL-1*β*: interleukin 1*β*, TGF-*β*: transforming growth factor *β*, and ICAM-1: intercellular adhesion molecule 1.

**Table 2:** Effect of ATG treatment on haematologic parameters in adult BALB/cOlaHsd mice (*n* = 3).

	ATG	Control
WBC (10/*µ*L)	705 ± 354	1434 ± 416
RBC (10^4^/*µ*L)	1041 ± 121	883 ± 176
Hb (g/L)	152 ± 21	137 ± 26
Hct (10^−1^%)	496 ± 45	415 ± 59
MCV (10^−1^ fL)	478 ± 33	473 ± 26
MCH (10^−1^ pg)	146 ± 6	155 ± 2
MCHC (g/L)	306 ± 22	328 ± 15
PLT (10^−3^/*µ*L)	1518 ± 238	1425 ± 289
NEUT (10/*µ*L)	282 ± 214	750 ± 384
LYM (10/*µ*L)	320 ± 110^**∗**^	676 ± 245
MONO (10/*µ*L)	91 ± 50	201 ± 105
EOS (10/*µ*L)	6.8 ± 4.2^**∗∗**^	25 ± 1
BAS (10/*µ*L)	5 ± 0.9^**∗∗∗**^	1.3 ± 1.2

A *t*-test was used to compare the means of the two samples: ^*∗*^ *p* = 0.07; ^*∗∗*^ *p* = 0.04; ^*∗∗∗*^ *p* = 0.004.

**Table 3:** Body weight, relative kidney weight, and renal function in BALB/cOlaHsd mice 4 days after cisplatin administration.

	Control	MSCs	ATG	ATG + MSCs	CIS	CIS + MSCs	ATG + CIS	ATG + CIS + MSCs
Body weight (g), day 0	27.3 ± 0.6	25.6 ± 1.2	27.6 ± 2.2	27.4 ± 2.7	24.9 ± 0.7	24.6 ± 0.8	26.6 ± 0.4	26.9 ± 0.3
Body weight change (g) (day 0–day 4)	0.9 ± 0.4^b^	0.2 ± 0.2^b^	0.3 ± 0.4^b^	0.5 ± 0.4^b^	(−) 5.1 ± 0.8^a^	(−) 4.8 ± 0.5^a^	(−) 4.8 ± 0.4^a^	(−) 4.5 ± 0.9^a^
Kidney relative weight ×100	1.68 ± 0.05^ab^	1.69 ± 0.06^ab^	1.67 ± 0.05^ab^	1.74 ± 0.05^ab^	1.71 ± 0.27^b^	1.67 ± 0.17^ab^	1.79 ± 0.19^b^	1.51 ± 0.06^a^
BUN (mmol/L)	8.05 ± 0.77^b^	7.43 ± 0.2^b^	8.4 ± 0.47^b^	6.95 ± 0.37^b^	43.87 ± 6.57^a^	42.7 ± 4.63^a^	40.26 ± 6.84^a^	31.34 ± 3.99^a^
Creatinine (*μ*mol/L)	30.5 ± 0.77^bc^	18.0 ± 0.2^bc^	18.25 ± 0.47^bc^	31.5 ± 7.63^bc^	83.67 ± 19.43^a^	48.0 ± 16.83^b^	42.0 ± 3.96^bc^	27.5 ± 1.91^c^

ANOVA followed by Duncun multicomparison test; results are expressed as mean ± SEM; values with different superscript letters in rows are statistically different (*p* < 0.05). ATG + CIS + MSCs: cisplatin treated with single injection of MSCs pretreated with ATG, ATG + CIS: cisplatin, pretreated with ATG, ATG + MSCs: treated with single injection of MSCs pretreated with ATG, ATG: treated with ATG, CIS + MSCs: cisplatin treated with single injection of MSCs, CIS: cisplatin treated, and MSCs: treated with single injection of MSCs.

## References

[1] Wang Y., He J., Pei X., Zhao W. (2013). Systematic review and meta-analysis of mesenchymal stem/stromal cells therapy for impaired renal function in small animal models. *Nephrology*.

[2] Shalaby R. H., Rashed L. A., Ismaail A. E., Madkour N. K., Elwakeel S. H. (2014). Hematopoietic stem cells derived from human umbilical cord ameliorate cisplatin-induced acute renal failure in rats. *American Journal of Stem Cells*.

[3] Morigi M., De Coppi P. (2014). Cell therapy for kidney injury: different options and mechanisms—mesenchymal and amniotic fluid stem cells. *Nephron—Experimental Nephrology*.

[4] Morigi M., Rota C., Montemurro T. (2010). Life-sparing effect of human cord blood-mesenchymal stem cells in experimental acute kidney injury. *Stem Cells*.

[5] Eliopoulos N., Zhao J., Bouchentouf M. (2010). Human marrow-derived mesenchymal stromal cells decrease cisplatin renotoxicity in vitro and in vivo and enhance survival of mice post-intraperitoneal injection. *American Journal of Physiology—Renal Physiology*.

[6] Fang T.-C., Pang C.-Y., Chiu S.-C., Ding D.-C., Tsai R.-K. (2012). Renoprotective effect of human umbilical cord-derived mesenchymal stem cells in immunodeficient mice suffering from acute kidney injury. *PLoS ONE*.

[7] Moghadasali R., Azarnia M., Hajinasrollah M. (2014). Intra-renal arterial injection of autologous bone marrow mesenchymal stromal cells ameliorates cisplatin-induced acute kidney injury in a rhesus *Macaque mulatta* monkey model. *Cytotherapy*.

[8] Imberti B., Morigi M., Benigni A. (2011). Potential of mesenchymal stem cells in the repair of tubular injury. *Kidney International Supplements*.

[9] Lee H. K., Lim S. H., Chung I. S. (2014). Preclinical efficacy and mechanisms of mesenchymal stem cells in animal models of autoimmune diseases. *Immune Network*.

[10] Baraniak P. R., McDevitt T. C. (2010). Stem cell paracrine actions and tissue regeneration. *Regenerative Medicine*.

[11] Castro-Manrreza M. E., Mayani H., Monroy-García A. (2014). Human mesenchymal stromal cells from adult and neonatal sources: a comparative in vitro analysis of their immunosuppressive properties against t cells. *Stem Cells and Development*.

[12] Wu K.-H., Chan C.-K., Tsai C. (2011). Effective treatment of severe steroid-resistant acute graft-versus-host disease with umbilical cord-derived mesenchymal stem cells. *Transplantation*.

[13] Lin C.-S., Lin G., Lue T. F. (2012). Allogeneic and xenogeneic transplantation of adipose-derived stem cells in immunocompetent recipients without immunosuppressants. *Stem Cells and Development*.

[14] Miller R. P., Tadagavadi R. K., Ramesh G., Reeves W. B. (2010). Mechanisms of cisplatin nephrotoxicity. *Toxins*.

[15] Liu M., Chien C.-C., Burne-Taney M. (2006). A pathophysiologic role for T lymphocytes in murine acute cisplatin nephrotoxicity. *Journal of the American Society of Nephrology*.

[16] Mohty M. (2007). Mechanisms of action of antithymocyte globulin: T-cell depletion and beyond. *Leukemia*.

[17] Watson M. J., Ke B., Shen X.-D. (2012). Treatment with antithymocyte globulin ameliorates intestinal ischemia and reperfusion injury in mice. *Surgery*.

[18] Wang H.-S., Hung S.-C., Peng S.-T. (2004). Mesenchymal stem cells in the Wharton's jelly of the human umbilical cord. *Stem Cells*.

[19] Dominici M., Le Blanc K., Mueller I. (2006). Minimal criteria for defining multipotent mesenchymal stromal cells. The International Society for Cellular Therapy position statement. *Cytotherapy*.

[20] Zhang J., Goering P. L., Espandiari P. (2009). Differences in immunolocalization of Kim-1, RPA-1, and RPA-2 in kidneys of gentamicin-, cisplatin-, and valproic acid-treated rats: potential role of iNOS and nitrotyrosine. *Toxicologic Pathology*.

[21] Jouan-Lanhouet S., Riquet F., Duprez L., Berghe T. V., Takahashi N., Vandenabeele P. (2014). Necroptosis, in vivo detection in experimental disease models. *Seminars in Cell and Developmental Biology*.

[22] Su Z., Yang Z., Xu Y., Chen Y., Yu Q. (2015). Apoptosis, autophagy, necroptosis, and cancer metastasis. *Molecular Cancer*.

[23] Lee H., Nho D., Chung H.-S. (2010). CD4^+^ CD25^+^ regulatory T cells attenuate cisplatin-induced nephrotoxicity in mice. *Kidney International*.

[24] Grigoryev D. N., Liu M., Hassoun H. T., Cheadle C., Barnes K. C., Rabb H. (2008). The local and systemic inflammatory transcriptome after acute kidney injury. *Journal of the American Society of Nephrology*.

[25] Meek R. L., Benditt E. P. (1986). Amyloid A gene family expression in different mouse tissues. *The Journal of Experimental Medicine*.

[26] Faubel S., Lewis E. C., Reznikov L. (2007). Cisplatin-induced acute renal failure is associated with an increase in the cytokines interleukin (IL)-1*β*, IL-18, IL-6, and neutrophil infiltration in the kidney. *Journal of Pharmacology and Experimental Therapeutics*.

[27] Ozkok A., Edelstein C. L. (2014). Pathophysiology of cisplatin-induced acute kidney injury. *BioMed Research International*.

[28] Ramesh G., Reeves W. B. (2002). TNF-*α* mediates chemokine and cytokine expression and renal injury in cisplatin nephrotoxicity. *The Journal of Clinical Investigation*.

[29] Kelly K. J., Williams W. W., Colvin R. B., Bonventre J. V. (1994). Antibody to intercellular adhesion molecule 1 protects the kidney against ischemic injury. *Proceedings of the National Academy of Sciences of the United States of America*.

[30] Pabla N., Dong Z. (2008). Cisplatin nephrotoxicity: mechanisms and renoprotective strategies. *Kidney International*.

[31] Ali B. H., Al Moundhri M. S. (2006). Agents ameliorating or augmenting the nephrotoxicity of cisplatin and other platinum compounds: a review of some recent research. *Food and Chemical Toxicology*.

[32] Agarwal A., Balla J., Alam J., Croatt A. J., Nath K. A. (1995). Induction of heme oxygenase in toxic renal injury: a protective role in cisplatin nephrotoxicity in the rat. *Kidney International*.

[33] Shiraishi F., Curtis L. M., Truong L. (2000). Heme oxygenase-1 gene ablation or expression modulates cisplatin-induced renal tubular apoptosis. *The American Journal of Physiology—Renal Physiology*.

[34] Erman A., Lakota K., Mrak-Poljsak K. (2012). Uropathogenic escherichia coli induces serum amyloid a in mice following urinary tract and systemic inoculation. *PLoS ONE*.

[35] Groh M. E., Maitra B., Szekely E., Koç O. N. (2005). Human mesenchymal stem cells require monocyte-mediated activation to suppress alloreactive T cells. *Experimental Hematology*.

[36] Uccelli A., Moretta L., Pistoia V. (2008). Mesenchymal stem cells in health and disease. *Nature Reviews Immunology*.

[37] Motaln H., Schichor C., Lah T. T. (2010). Human mesenchymal stem cells and their use in cell-based therapies. *Cancer*.

[38] Motaln H., Turnsek T. L. (2015). Cytokines play a key role in communication between mesenchymal stem cells and brain cancer cells. *Protein & Peptide Letters*.

